# Effects of Tanreqing injection against ventilator-associated pneumonia: a meta-analysis and systematic review of clinical studies

**DOI:** 10.3389/fphar.2025.1545088

**Published:** 2025-03-07

**Authors:** Pochen Li, Yang Wu, Danxia Ge, Ruyi Xu, Qianping Zhang, Yujiao Li, Lingyao Zhang, HsuanChieh Peng, Fangyu Yu

**Affiliations:** ^1^ Intensive Care Unit, Ningbo Municipal Hospital of Traditional Chinese Medicine (TCM), Affiliated Hospital of Zhejiang Chinese Medical University, Ningbo, China; ^2^ Department of Respiratory, Ningbo Municipal Hospital of Traditional Chinese Medicine (TCM), Affiliated Hospital of Zhejiang Chinese Medical University, Ningbo, China; ^3^ Department of Respiratory, Dongzhimen Hospital, Beijing University of Chinese Medicine, Beijing, China

**Keywords:** tanreqing injection, ventilator-associated pneumonia, meta-analysis, systematic review, traditional chinese medicine, botanical drugs

## Abstract

**Objective:**

Ventilator-associated pneumonia (VAP) frequently results in difficulties with weaning, high mortality rates, and is often caused by drug-resistant pathogens, emphasizing the critical importance of effective treatment. The efficacy and safety of Tanreqing injection (TRQI) in the treatment of VAP patients have been demonstrated, but further validation is required. The objective of this study is to synthesize the findings of clinical research on TRQI for the treatment of VAP, thereby providing clinical evidence for its effectiveness and importance.

**Methods:**

A comprehensive search of eight databases was conducted, covering all records up to 30 August 2024. The data were extracted, quality-assessed, and analyzed rigorously. The methodological quality of the included studies was evaluated using the RoB-2 tool. The statistical analyses were conducted using RevMan 5.4 software, with either a fixed-effect or random-effect model employed as appropriate. The evidence quality of the included literature was evaluated using Grade pro 3.6.1 software.

**Results:**

A total of 20 clinical studies, comprising a total of 1,446 patients, were included in the review. The meta-analysis of these studies demonstrated that TRQI significantly improved inflammatory markers (CRP, PCT, WBC) (*P* < 0.00001) and reduced the duration of antibiotic use (*P* < 0.00001). Furthermore, the intervention resulted in a shorter duration of ventilator usage (*P* < 0.0001), an increased initial weaning success rate (*P* = 0.001), and a reduction in the length of stay in the intensive care unit (ICU) (*P* < 0.00001). Furthermore, the TRQI demonstrated superior performance compared to the control group in CPIS (Clinical Pulmonary Infection Score) assessments (*P <* 0.00001). Meanwhile, the quality of evidence for CRP, PCT, Duration of Antibiotic Use, Duration of Ventilator Use, and Length of ICU Stay is Moderate.

**Conclusion:**

This study provides further evidence-based support for the clinical application of TRQI in the treatment of VAP. Additionally, it summarizes previous clinical research through a literature quality assessment, offering insights and recommendations for the design and implementation of future research protocols. The findings indicate that TRQI can improve inflammatory markers and pulmonary infection scores in VAP patients, reduce ventilator dependence, and shorten antibiotic use duration. Moreover, it has a low overall incidence of adverse reactions, demonstrating good efficacy and safety as an adjuvant therapy for VAP. However, some of the included clinical studies had limitations such as small sample sizes, lack of sample size calculations. Therefore, future study designs should be more rigorous to enhance the reliability of findings.

**Systematic Review Registration:**

https://inplasy.com/inplasy-2025-2-0008/.

## 1 Introduction

Ventilator-associated pneumonia (VAP) is a type of nosocomial pneumonia that develops after a minimum of 48 h of mechanical ventilation following endotracheal intubation (Metersky and Kalil, 2024). Patients with VAP frequently exhibit compromised immune function, complex underlying diseases, severe infections, and multiple organ dysfunction. The prolonged and excessive use of antibiotics in these patients can result in the rapid development of drug-resistant pathogens, thereby reducing the clinical efficacy of the treatment (Papazian et al., 2020). Consequently, due to the impact of pathogen resistance and the patient’s physical condition, VAP often results in extended mechanical ventilation and antibiotic use during routine treatment, lower weaning success rates, prolonged ICU stays, increased risk of in-hospital mortality, and a higher socioeconomic burden on patients (Torres et al., 2017).

Clinical evaluation of the efficacy of VAP can be conducted through imaging studies, inflammatory markers, and changes in symptoms and signs. Among them, the Clinical Pulmonary Infection Score (CPIS) is a commonly used clinical indicator for assessing nosocomial pulmonary infections. The CPIS has a total score of 12 points and comprises the following components: degree of fever, white blood cell count, oxygenation status, extent of pulmonary infiltrates on chest radiography, and characteristics of airway secretions. Simultaneously, inflammatory biomarkers are widely used in clinical practice due to their objectivity and direct interpretability. Common inflammatory markers include white blood cell count (WBC), C-reactive protein (CRP), procalcitonin (PCT), and interleukin-6 (IL-6). WBC is often significantly elevated in various infectious diseases and serves as an indicator of the body’s inflammatory response. CRP, a non-specific protein synthesized by the liver, begins to rise within 6–8 h of an acute infection, providing support for the early diagnosis of inflammatory diseases. Additionally, CRP levels exhibit a clear positive correlation with infection severity, making it a useful reference for clinical efficacy assessment. PCT, a hormone-inactive glycoprotein produced by thyroid C cells, has a certain degree of specificity for bacterial infections. Its levels also correlate positively with the severity of infection, making it a valuable biomarker for evaluating treatment response and guiding antibiotic use.

Tanreqing Injection (TRQI) is a traditional Chinese medicine (TCM) extract injection that was granted a Chinese drug approval number in 2003, with the national drug approval code Z20030054. The manufacturing process of this drug is well-established, enabling large-scale production while ensuring product quality stability and consistency. The production process complies with the Chinese national drug standard YBZ00912003-2007Z-2009 and has passed relevant quality certifications. TRQI is composed of five medicinal ingredients: Lian-qiao (*Forsythia suspensa* (*Thunb.*) *Vahl* [Oleaceae; *Forsythiae Fructus*]), Jin-yin-hua (*Lonicera japonica Thunb.* [*Caprifoliaceae*; *Lonicerae Japonicae Flos*]), Huang-qin (*Scutellaria baicalensis Georgi* [*Lamiaceae*; *Scutellariae Baicalensis Radix*]), Antelope horn (*Saiga tatarica Linnaeus* [*Bovidae*; *Saigae Tataricae Cornu*]), and bear bile powder (*Ursus thibetanus Cuvier* [*Ursidae*; *Fel Ursi*] or *Ursus arctos Linnaeus* [*Ursidae*; *Fel Ursi*]). In TCM, it is known for its effects in clearing heat, resolving phlegm, detoxifying, and restoring consciousness. Biomedically, it exhibits pharmacological properties such as anti-inflammatory, antitussive, antibacterial, and bronchodilatory effects (Wang et al., 2020). *In vitro* studies have demonstrated that TRQI exerts considerable inhibitory effects on a range of Gram-negative and Gram-positive bacteria, including *Streptococcus pneumoniae*, *Streptococcus hemolyticus*, and *Staphylococcus aureus* (Cui et al., 2023). Furthermore, it effectively modulates inflammatory responses in patients (Liu et al., 2016).

In TCM, VAP is classified under the category of “lung inflammation with wheezing and coughing.” It is postulated that patients undergoing mechanical ventilation frequently exhibit deficiencies in vital energy (Zheng Qi), rendering them vulnerable to external pathogenic heat. This heat penetrates inward, disrupting lung function and leading to the accumulation of phlegm-heat and airway obstruction, which manifests as coughing, sputum production, shortness of breath, and fever. Accordingly, TCM places emphasis on the principles of clearing heat, resolving phlegm, promoting lung function, and relieving cough as the primary treatment approach for VAP.

In recent years, a considerable number of scholars from China and internationally have conducted a series of randomized controlled trials (RCTs) to investigate the efficacy of TRQI in the treatment of VAP. To provide a more robust evidence base, this study conducted a comprehensive search of relevant databases and selected eligible RCTs for a meta-analysis, thereby offering support for future clinical medication practices.

## 2 Materials and methods

### 2.1 Pre-register protocol

This study has been registered on the International Platform of Registered Systematic Review and Meta-analysis Protocols (Inplasy) under the registration number: INPLASY202520008, DOI number: 10.37766/inplasy 2025.2.0008. This study was conducted in strict accordance with the registration protocol.

### 2.2 Search strategy and selected databases

A systematic search was conducted in the following databases: the US National Library of Medicine’s PubMed, the Web of Science, Embase, The Cochrane Library, CNKI, WanFang Data, VIP, and SinoMed. The objective was to identify RCTs on TRQI for the treatment of VAP. The search period spanned the inception of each database up to 30 August 2024. In order to identify eligible studies, both subject terms and free-text terms were employed, and a manual search was conducted. The search was limited to literature in English and Chinese. The detailed search strategies and results from the eight databases are provided in the Supplementary Material.

### 2.3 Inclusion and exclusion criteria

#### 2.3.1 Inclusion criteria

1) Study Type: RCTs were included in the study.
2) The study population was as follows: Patients who have been clinically diagnosed with VAP in clinical studies.
3) Intervention Measures: In the experimental group, the intervention involved the administration of routine treatment (RT) in conjunction with TRQI, whereas the control group received either RT alone or RT in conjunction with a placebo.
4) Outcome Indicators: Outcome measures included: (i) CRP; (ii) PCT; (iii) WBC count; (iv) Duration of ventilator use; (v) Duration of antibiotic use; (vi) First successful weaning rate; (vii) Length of ICU stay; (viii) CPIS; (ix) Adverse drug reactions.

#### 2.3.2 Exclusion criteria

1) Studies involving patients with community-acquired pneumonia (CAP);
2) Duplicate studies, reviews, clinical protocols, commentaries, and case reports;
3) Studies that include interventions involving other traditional Chinese medicines or related therapies in addition to TRQI;
4) Studies where data could not be obtained, even after contacting the original authors;
5) Studies without a control group;
6) Studies that are duplicate publications;
7) Studies with a sample size of fewer than 40 participants.

### 2.4 Data extraction

The following information was extracted from the final included studies by independent reviewers: (i) name of the first author and year of publication; (ii) specific details of clinical patients in each study, including age, sample size, and intervention measures; (iii) outcome indicators, including clinical efficacy and adverse drug reactions. In the event that a study encompassed multiple observation time points, the results from the final time point were recorded.

### 2.5 Risk of bias in included studies

The quality of the clinical studies was evaluated using the RoB-2 tool, which assesses the following domains: The following domains were assessed for each study: 1) Randomization process; 2) Bias due to deviations from intended interventions; 3) Bias in adherence to interventions; 4) Missing outcome data; 5) Bias in measurement of outcomes; 6) Selective reporting of results; 7) Overall RoB-2 score (Sterne et al., 2019). Two reviewers conducted an independent assessment of the quality of the studies. In the event of a discrepancy, discussions were held or the corresponding author was consulted to reach a resolution.

### 2.6 Statistical analysis

The statistical analysis was conducted using RevMan 5.4 software. The I^2^ test was employed for the purpose of assessing heterogeneity. In accordance with the guidelines set forth in the Cochrane Handbook for Systematic Reviews of Interventions (Higgins et al., 2019), I^2^ values of 0%–40% may be indicative of low heterogeneity, 30%–60% may indicate moderate heterogeneity, 50%–90% may be indicative of substantial heterogeneity, and 75%–100% may indicate considerable heterogeneity. In instances where the I^2^ value fell below 50%, a fixed-effects model was employed. Conversely, when the I^2^ value reached or exceeded 50%, a random-effects model was utilized. A p-value of less than 0.05 was deemed statistically significant. In the case of categorical data, the relative risk (RR) was employed as the effect measure. In the case of continuous data, the mean difference (MD) was employed when measurement methods and units were consistent. Conversely, when such consistency was lacking, the standardized mean difference (SMD) was utilized when different measurement methods or units were involved.

### 2.7 Sensitivity analysis

A sensitivity analysis was conducted on the study data to indirectly identify the sources of heterogeneity and assess the stability and reliability of the pooled results. Based on the types and number of included studies, this study employed the ‘leave-one-out’ method, sequentially excluding each included study sample and observing changes in heterogeneity and meta-analysis results to evaluate the stability of the data.

### 2.8 Publication bias

The potential for publication bias for the primary outcomes is evaluated through the use of a funnel plot. The funnel plot is generated using RevMan 5.4 software.

### 2.9 GRADE assessment

The evidence quality of the included literature was evaluated using Grade pro 3.6.1 software. The quality of evidence was downgraded based on five aspects: risk of bias, inconsistency, indirectness, imprecision, and publication bias. Conversely, the quality of evidence was upgraded based on three aspects: effect size, confounding factors that may reduce the effect, and dose-response relationship. Since all the included studies in this research were RCTs, the initial evidence quality was rated as high, and thus, the focus was primarily on the downgrading criteria.

## 3 Results

### 3.1 Included studies

**FIGURE 1:**
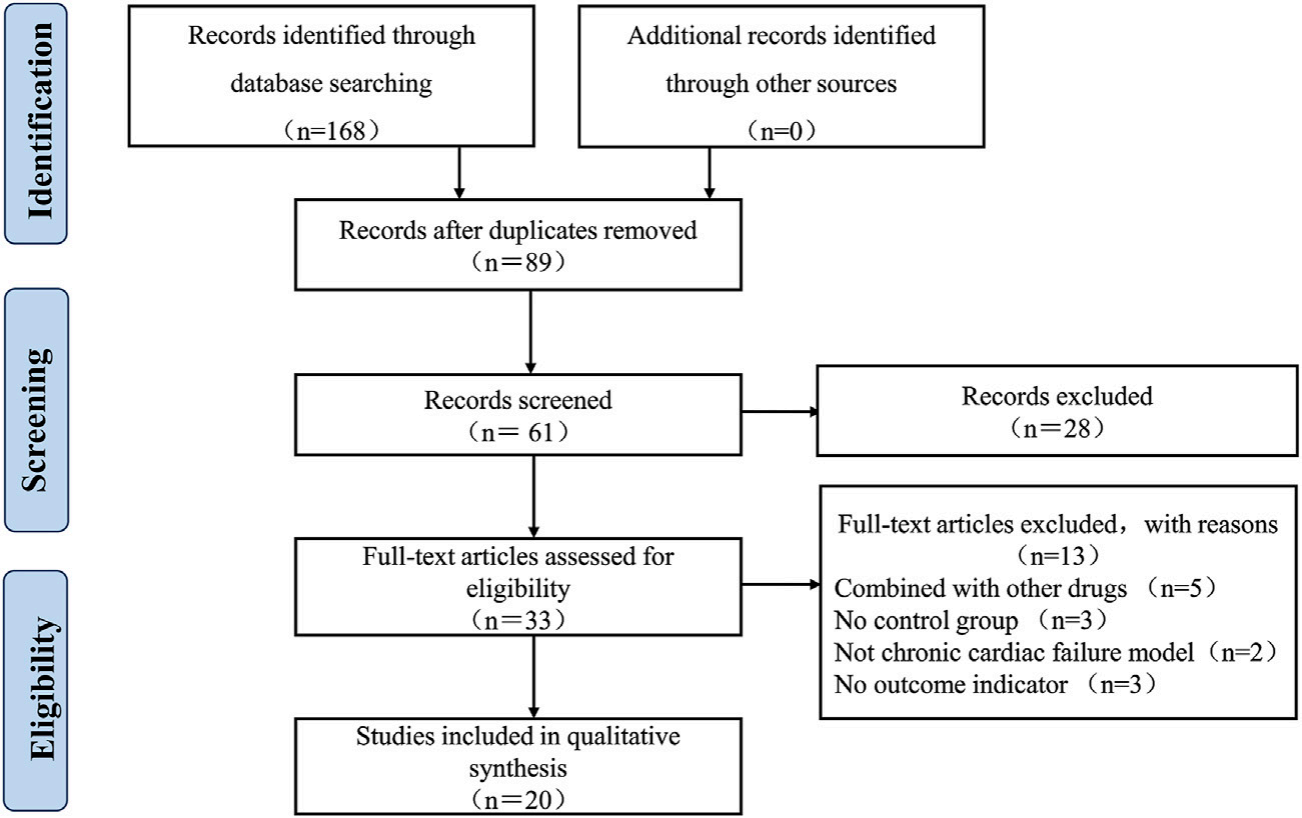
Literature screening process and results.

A total of 168 articles were retrieved from eight databases. Following the removal of duplicates using Endnote X9 software, 89 articles were retained. Two reviewers conducted independent screening of titles and abstracts, resulting in the narrowing of the selection to 33 articles. Subsequently, both reviewers conducted a comprehensive review of the full texts of the 33 articles, resulting in the inclusion of 20 clinical studies published between 2009 and 2023. Any discrepancies that arose during the selection process were resolved by corresponding author (Figure 1).

### 3.2 Basic characteristics of included studies

The 20 included clinical trials (Wang, 2015; Chen and Xi, 2015; Chen and Hu, 2015; He, 2018; Hou et al., 2015; Hu, 2017; Huang, 2014; Lang, 2015; Liao et al., 2023; Liu and Liang, 2016; Mao et al., 2014; Mi, 2016; Miao et al., 2009; Pan et al., 2013; Tian and Yang, 2012; Wang et al., 2022; Wang and Wang, 2018; Yu and Liu, 2009; Zhang and Li, 2017; Zhang et al., 2012) had sample sizes ranging from 40 to 155 participants. The experimental group received a combination of TRQI and RT, while the control group underwent RT alone. The dosage of TRQI ranged from 20 to 40 mL per day, with treatment durations of one to 2 weeks. The included outcome indicators were CRP, PCT, WBC count, duration of antibiotic use, duration of ventilator use, first successful weaning rate, length of ICU stay, CPIS score, and incidence of adverse drug reactions (Table 1).

**TABLE 1:** Basic characteristics of the 20 clinical studies.

Study ID	Sample size (T/C)	Mean age (years)	T	C	Usage and dosage	Duration	Outcomes
Liao X 2023	78 (39/39)	58.34 ± 6.91/57.63 ± 6.72	RT + TRQI	RT	20 mL qd	2w	3, 4
Wang SL 2022	63 (32/31)	5.69 ± 1.48/63.81 ± 6.61	RT + TRQI	RT	20 mL qd	2w	4, 7, 9
Wang HT 2018	56 (28/28)	44.24 ± 2.24/45.05 ± 1.64	RT + TRQI	RT	20 mL qd	2w	2
He JT 2018	60 (30/30)	64.1 ± 5.0/64.3 ± 5.5	RT + TRQI	RT	20 mL qd	1w	1, 2, 3
Hu CJ 2017	66 (/33/33)	49.67 ± 2.63/48.76 ± 2.82	RT + TRQI	RT	20 mL qd	2w	4, 5, 6
Zhang LJ 2017	84 (42/42)	73 ± 18/70 ± 13	RT + TRQI	RT	20 mL qd	1w	3, 7
Mi J 2016	100 (50/50)	61.5 ± 9.7/61.3 ± 9.4	RT + TRQI	RT	20 mL qd	2w	4, 5, 6
Liu R 2016	88 (44/44)	62.48 ± 8.27/63.14 ± 7.95	RT + TRQI	RT	20 mL qd	2w	1, 2, 3
Chen YG 2015	40 (20/20)	60.1 ± 13.8/59.3 ± 13.6	RT + TRQI	RT	40 mL qd	1w	1, 2, 3, 6, 7
Lang B 2015	74 (37/37)	60 ± 4	RT + TRQI	RT	20 mL qd	10d	2
Chen JM 2015	96 (51/45)	72.57 ± 16.21/70.68 ± 17.04	RT + TRQI	RT	20 mL qd	10d	2, 3, 6, 7, 8
Hou XZ 2015	72 (36/36)	63.2 ± 9.5/62.6 ± 9.2	RT + TRQI	RT	20 mL qd	2w	4, 5, 6, 9
Wang HR 2015	60 (30/30)	58. 9 ± 10. 8	RT + TRQI	RT	20 mL qd	1w	2, 3, 7
Huang GR2014	62 (31/31)	64.5 ± 1.0/67.0 ± 0.5	RT + TRQI	RT	20 mL qd	2w	2, 5, 6
Mao ZF 2014	66 (33/33)	63.44 ± 5.33/63.28 ± 4.67	RT + TRQI	RT	20 mL qd	2w	1, 2, 3, 4, 5, 6
Pan LN 2013	56 (28/28)	68.5 ± 1.3/65.4 ± 1.2	RT + TRQI	RT	20 mL qd	10d	2
Zhang X 2012	62 (31/31)	68.01 ± 1.30/65.00 ± 1.40	RT + TRQI	RT	20 mL qd	10d	1, 8
Tian J 2012	58 (30/28)	62.5 ± 10.6/61.7 ± 9.7	RT + TRQI	RT	20 mL qd	2w	1, 2, 3, 4, 5, 6
Yu YX 2009	155 (81/74)	56.31 ± 27.02/55.49 ± 25.83	RT + TRQI	RT	20 mL qd	1w	2, 8
Miao LX 2009	50 (25/25)	55.25 ± 9.12	RT + TRQI	RT	20 mL qd	10d	8

Note: T: treatment group; C: control group; TRQI: tanreqing injection; RT: routine treatment; 1. WBC, count; 2. CRP; 3. PCT; 4. Duration of antibiotic use; 5. Length of ICU, stay; 6. Duration of ventilator use; 7. CPIS, score; 8. First successful weaning rate; 9. Adverse reactions.

### 3.3 Risk of bias results

All RCTs were determined to have a low risk of bias with adhering to intervention. With bias in randomization process, assignment to intervention, missing outcome data, measurement of outcome, and bias due to Selection of the reported result, some studies were deemed to have a low risk, while others were identified as having some risk (Table 2).

**TABLE 2:** Methodological evaluation of included clinical studies (RoB-2 method).

Study ID	Randomization process	Assignment to intervention	Adhering to intervention	Missing outcome data	Measurement of outcome	Selection of the reported result	Overall score
Liao X 2023	Low risk	Some concerns	Low risk	Low risk	Some concerns	Some concerns	3
Wang SL 2022	Low risk	Some concerns	Low risk	Low risk	Low risk	Some concerns	4
Wang HT 2018	Some concerns	Some concerns	Low risk	Low risk	Some concerns	Some concerns	2
He JT 2018	Low risk	Low risk	Low risk	Low risk	Some concerns	Some concerns	4
Hu CJ 2017	Some concerns	Some concerns	Low risk	Low risk	Some concerns	Some concerns	2
Zhang LJ 2017	Low risk	Some concerns	Low risk	Low risk	Low risk	Some concerns	4
Mi J 2016	Low risk	Low risk	Low risk	Low risk	Low risk	Some concerns	5
Liu R 2016	Some concerns	Some concerns	Low risk	Low risk	Low risk	Some concerns	3
Chen YG 2015	Low risk	Some concerns	Low risk	Low risk	Some concerns	Some concerns	3
Lang B 2015	Low risk	Some concerns	Low risk	Low risk	Low risk	Some concerns	4
Chen JM 2015	Some concerns	Low risk	Low risk	Low risk	Some concerns	Some concerns	3
Hou XZ 2015	Some concerns	Low risk	Low risk	Low risk	Some concerns	Some concerns	3
Wang HR 2015	Some concerns	Some concerns	Low risk	Low risk	Low risk	Some concerns	3
Huang GR 2014	Low risk	Low risk	Low risk	Low risk	Some concerns	Some concerns	4
Mao ZF 2014	Some concerns	Some concerns	Low risk	Low risk	Low risk	Some concerns	3
Pan LN 2013	Low risk	Some concerns	Low risk	Low risk	Some concerns	Some concerns	3
Zhang X 2012	Some concerns	Some concerns	Low risk	Low risk	Low risk	Some concerns	3
Tian J 2012	Some concerns	Low risk	Low risk	Low risk	Some concerns	Some concerns	3
Yu YX 2009	Low risk	Some concerns	Low risk	Some concerns	Some concerns	Some concerns	2
Miao LX 2009	Some concerns	Low risk	Low risk	Low risk	Low risk	Some concerns	4

### 3.4 Meta-analysis results

#### 3.4.1 CRP

**FIGURE 2:**
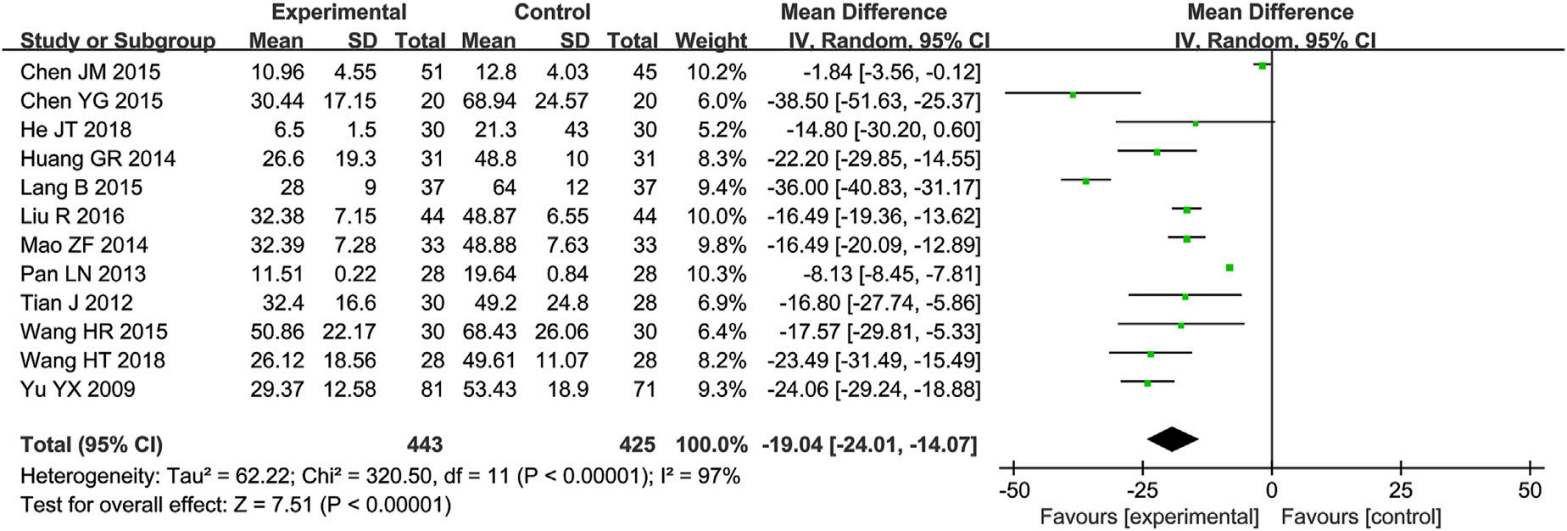
Meta-analysis results of CRP.

A total of 12 RCTs (Wang, 2015; Chen and Xi, 2015; Chen and Hu, 2015; He, 2018; Huang, 2014; Lang, 2015; Liu and Liang, 2016; Mao et al., 2014; Pan et al., 2013; Tian and Yang, 2012; Wang and Wang, 2018; Yu and Liu, 2009) reporting CRP values were included in the meta-analysis, involving 868 patients. A heterogeneity test was conducted. The I^2^ statistic yielded a value of 97%, indicating substantial heterogeneity. Consequently, a random-effects model was employed. The pooled effect size is as follows: The MD was −19.04 [95% confidence interval (CI): −24.01, −14.07], with a P < 0.00001, indicating statistically significant differences. Interpretation: The 95% confidence interval is to the left of 0, indicating that compared to the biomedical control group, the treatment group exhibited a statistically significant reduction in CRP levels. This demonstrates that the treatment group was more effective in improving CRP levels and reducing inflammatory responses (Figure 2).

#### 3.4.2 PCT

**FIGURE 3:**
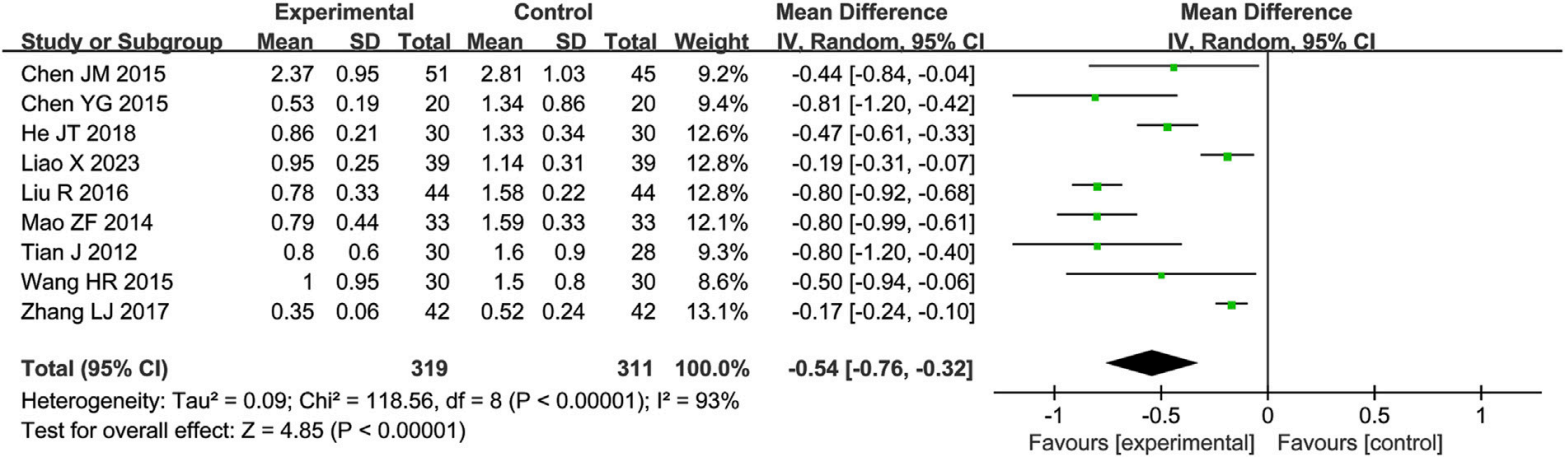
Meta-analysis results of PCT.

A total of nine RCTs (Wang, 2015; Chen and Xi, 2015; Chen and Hu, 2015; He, 2018; Liao et al., 2023; Liu and Liang, 2016; Mao et al., 2014; Tian and Yang, 2012; Zhang and Li, 2017) reporting PCT values were included in the analysis, involving 630 patients. A heterogeneity test was conducted. The I^2^ statistic yielded a value of 93%, indicating substantial heterogeneity. Consequently, a random-effects model was employed to account for this variability. The pooled effect size is as follows: The MD was −0.54 [95% CI: −0.76, −0.32], with a *P* < 0.00001, indicating a statistically significant difference. Interpretation: The 95% confidence interval is to the left of 0, indicating that compared to the biomedical control group, the treatment group exhibited a statistically significant reduction in PCT levels. This demonstrates that the treatment group was more effective in improving PCT levels (Figure 3).

#### 3.4.3 WBC

**FIGURE 4:**
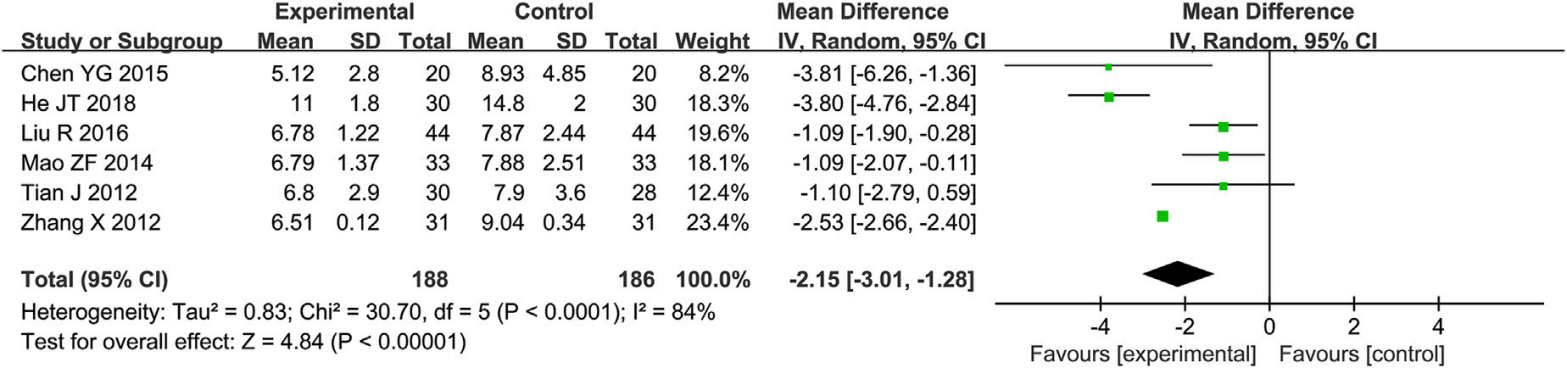
Meta-analysis results of WBC.

A total of six RCTs (Chen and Hu, 2015; He, 2018; Liu and Liang, 2016; Tian and Yang, 2012; Zhang et al., 2012) reporting WBC values were included in the analysis, involving a total of 374 patients. A heterogeneity test was conducted. The I^2^ statistic yielded a value of 84%, indicating substantial heterogeneity. Consequently, a random-effects model was employed. The pooled effect size is as follows: The MD was −2.15 [95% CI: −3.01, −1.28], with a *P* < 0.00001, indicating a statistically significant difference. Interpretation: The 95% confidence interval is to the left of 0, indicating that compared to the biomedical control group, the treatment group exhibited a statistically significant reduction in WBC count. This suggests that the treatment group was more effective in reducing white blood cell count, which may be related to the improvement of the patients’ inflammatory status (Figure 4).

#### 3.4.4 Duration of antibiotic use

**FIGURE 5:**
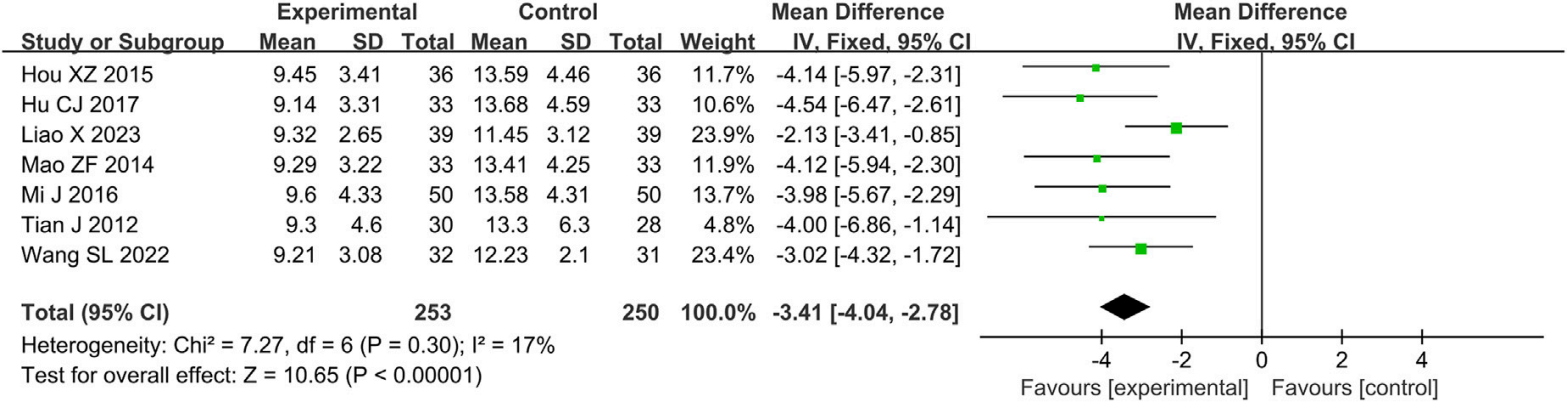
Meta-analysis results of duration of antibiotic use.

A total of seven RCTs (Hou et al., 2015; Hu, 2017; Liao et al., 2023; Liu and Liang, 2016; Mi, 2016; Tian and Yang, 2012; Wang et al., 2022) reporting the duration of antibiotic use were included in the analysis, involving a total of 503 patients. A heterogeneity test was conducted. The I^2^ statistic yielded a value of 17%, indicating low heterogeneity. Consequently, a fixed-effects model was employed. The pooled effect size is as follows: The MD was −3.41 [95% CI: −4.04, −2.78], with a *P* < 0.00001, indicating statistically significant differences. Interpretation: The 95% confidence interval is to the left of 0, indicating that compared to the biomedical control group, the treatment group exhibited a statistically significant reduction in antibiotic usage duration. This suggests that the treatment group was more effective in reducing antibiotic usage time, minimizing antibiotic application, and shortening the course of the disease (Figure 5).

#### 3.4.5 Duration of ventilator use

**FIGURE 6:**
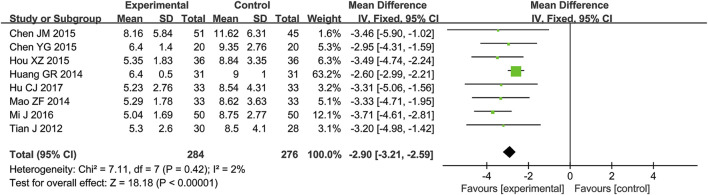
Meta-analysis results of duration of ventilator use.

A total of eight RCTs (Chen and Xi, 2015; Chen and Hu, 2015; Hou et al., 2015; Hu, 2017; Huang, 2014; Liu and Liang, 2016; Mi, 2016; Tian and Yang, 2012) reporting the duration of ventilator use were included in the analysis, involving 560 patients. A heterogeneity test was conducted. The I^2^ statistic yielded a value of 2%, indicating minimal heterogeneity. Consequently, a fixed-effects model was employed. The pooled effect size is as follows: The MD was −2.90 [95% CI: −3.21, −2.59], with a *P* < 0.00001, indicating a statistically significant difference. Interpretation: The 95% confidence interval is to the left of 0. It indicates that, compared to the biomedical control group, the treatment group exhibited a statistically significant reduction in ventilator usage duration. This suggests that the treatment group was more effective in shortening mechanical ventilation time and preventing ventilator dependence (Figure 6).

#### 3.4.6 Length of ICU stay

**FIGURE 7:**
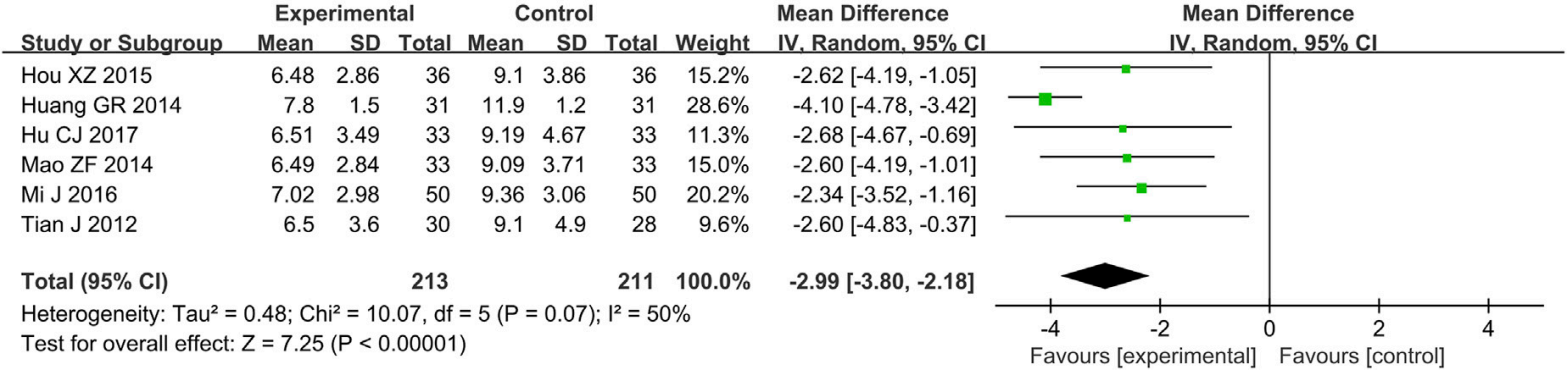
Meta-analysis results of length of ICU stay.

A total of six RCTs (Hou et al., 2015; Hu, 2017; Huang, 2014; Mao et al., 2014; Mi, 2016; Tian and Yang, 2012) reporting the length of ICU stay were included in the analysis, involving 424 patients. A heterogeneity test was conducted. The I^2^ statistic yielded a value of 50%, indicating moderate heterogeneity. Consequently, a random-effects model was employed. The pooled effect size is as follows: The MD was −2.99 [95% CI: −3.80, −2.18], with a *P* < 0.00001, indicating a statistically significant difference. Interpretation: The 95% confidence interval is to the left of 0. The above results indicate that, compared to the biomedical control group, the treatment group exhibited a statistically significant reduction in ICU length of stay. This suggests that the treatment group was more effective in improving the patient’s condition (Figure 7).

#### 3.4.7 First successful weaning rate

**FIGURE 8:**
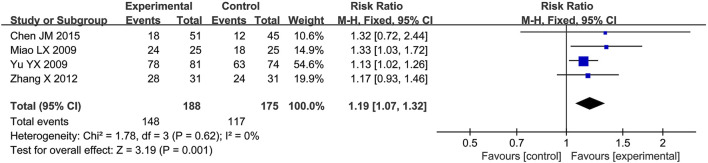
Meta-analysis results of first successful weaning rate.

A total of four RCTs (Chen and Xi, 2015; Miao et al., 2009; Yu and Liu, 2009; Zhang et al., 2012) reporting the first successful weaning rate were included in the analysis, involving a total of 363 patients. A heterogeneity test was conducted. The I^2^ statistic yielded a value of 0%, indicating the absence of heterogeneity. Consequently, a fixed-effects model was employed. The pooled effect size is as follows: The RR value was 1.19 [95% CI: 1.07, 1.32], with *P* = 0.001, indicating statistically significant differences. Interpretation: The 95% confidence interval is to the right of 0. The results indicate that, compared to the biomedical control group, the treatment group exhibited a statistically significant increase in the first successful weaning rate. This suggests that the treatment group was more effective in reducing ventilator dependence, and enhancing prognosis (Figure 8).

#### 3.4.8 CPIS score

**FIGURE 9:**
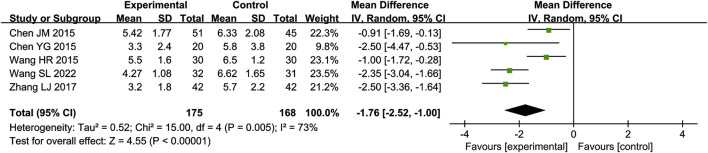
Meta-analysis results of CPIS score.

A total of five RCTs (Wang, 2015; Chen and Xi, 2015; Chen and Hu, 2015; Wang et al., 2022; Zhang and Li, 2017) reporting CPIS scores were included in the analysis, involving a total of 343 patients. A heterogeneity test was conducted. The I^2^ statistic yielded a value of 73%, indicating substantial heterogeneity. Consequently, a random-effects model was employed. The pooled effect size is as follows: The MD was −1.76 [95% CI: −2.52, −1.00], with a *P* < 0.00001, indicating statistically significant differences. Interpretation: The 95% confidence interval is to the left of 0. The results indicate that, compared to the biomedical control group, the treatment group exhibited a statistically significant reduction in CPIS scores. This suggests that the treatment group was more effective in improving patients’ pulmonary infections and alleviating symptoms and signs (Figure 9).

#### 3.4.9 Adverse reactions

A total of tow RCTs (Hou et al., 2015; Wang et al., 2022) reporting adverse reactions were included in the analysis, involving a total of 135 patients. In one study (Hou et al., 2015), all 72 patients included did not experience any adverse events. In the other study (Wang et al., 2022), among the 63 patients, both the experimental and control groups reported 6 cases of adverse reactions, primarily rash and nausea. No severe adverse reactions were observed.

### 3.5 Sensitivity analysis

To assess the impact of individual studies on the heterogeneity of outcome indicators with high heterogeneity (CRP, PCT, WBC, length of ICU stay, and CPIS score), each study was excluded one by one. Nevertheless, the heterogeneity of these indicators remained unaltered. This suggests that the results demonstrate strong reliability and stability.

### 3.6 Publication bias

**FIGURE 10:**
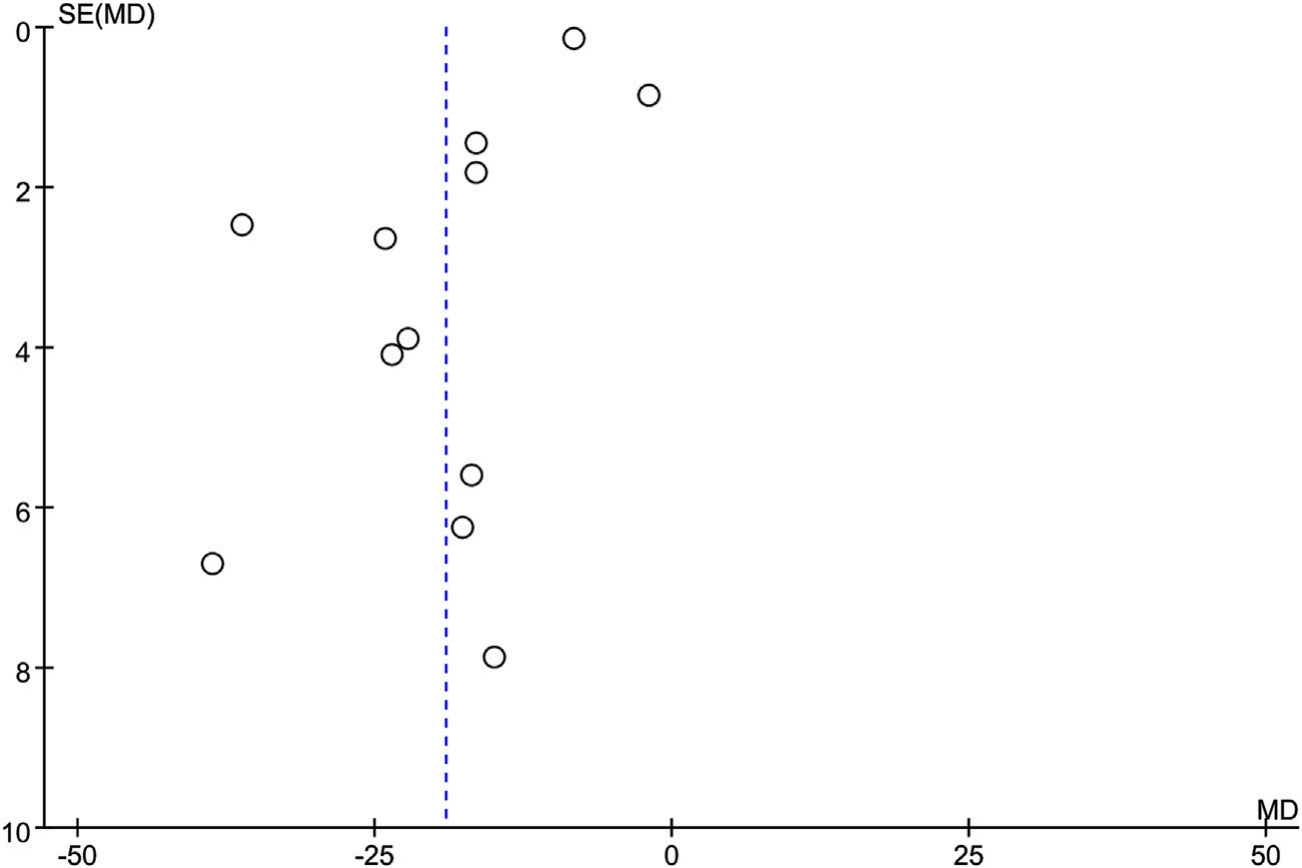
Funnel plot.

A funnel plot was employed to assess publication bias, with CRP serving as the outcome indicator based on the 12 included studies. The resulting funnel plot demonstrated that the points were not entirely symmetrically distributed on either side of the midline, indicating the potential presence of publication bias in the included studies (Figure 10).

### 3.7 GRADE assessment

The GRADE assessment results indicate that the quality of evidence for CRP, PCT, Duration of Antibiotic Use, Duration of Ventilator Use, and Length of ICU Stay is Moderate, while the quality of evidence for WBC, First Successful Weaning Rate, and CPIS Score is Low. The detailed results of the GRADE assessment have been provided in the Supplementary Material.

## 4 Discussion

### 4.1 Study findings

The present study encompasses a total of 20 clinical studies, involving a total of 1,446 patients. A systematic evaluation of the efficacy and safety of TRQI as an adjuvant therapy for VAP was conducted. The results of the clinical studies demonstrated that TRQI can improve inflammatory markers (CRP, PCT, WBC), reduce the duration of antibiotic use, ventilator use, and ICU stay, increase the first successful weaning rate, and improve CPIS scores. All of these differences were statistically significant. In terms of evidence quality, CRP, PCT, Duration of Antibiotic Use, Duration of Ventilator Use, and Length of ICU Stay are classified as Moderate. In addition, the limited sample size precluded a comprehensive statistical analysis of TRQI’s safety. However, according to the results reported in the original literature, its adverse reactions were mainly mild allergic and gastrointestinal reactions, and no serious adverse reactions were observed.

According to the drug’s instruction manual, a real world study involving 30,322 patients reported an adverse reaction incidence rate of approximately 0.27% (ClinicalTrials.gov ID NCT02094638). The main adverse reactions were occasional allergic responses, including dizziness, nausea, vomiting, generalized flushing, pruritus, or rash. To better understand the safety of TRQI, we reviewed relevant literature and found a post-marketing safety re-evaluation study indicated that the overall incidence of adverse drug reactions (ADRs) for TRQI was 0.17%, with vasculitis being the primary adverse reaction, and no severe ADRs were reported (Xu et al., 2016). Nevertheless as a TCM extract injection, there remains a risk of severe allergic reactions in clinical practice. According to the recommendations in the drug’s instruction manual, a detailed inquiry into the patient’s medication and allergy history should be conducted before administration. Patients should be closely monitored during administration, particularly within the first 5–30 min, and if an allergic reaction or other ADR occurs, the medication should be discontinued immediately, and symptomatic treatment should be provided. It is also crucial to adhere strictly to the recommended dosage and administration guidelines, including dilution requirements and infusion speed. Regarding drug interactions, TRQI should not be mixed with acidic component-containing injections. It has known incompatibilities with cefoperazone-sulbactam, pazufloxacin, amikacin, azithromycin, pantoprazole injection, calcium gluconate, vitamin B6, fructose-1,6-diphosphate, ambroxol hydrochloride, and cimetidine.

### 4.2 Potential mechanisms of TRQI in the treatment of VAP

#### 4.2.1 Efficacy and pharmacological substance of TRQI

TRQI contains various substances that have inhibitory effects on pathogenic microorganisms and regulate the release of inflammatory factors (Wang et al., 2020; Qiang, 2018; Liu et al., 2016; He et al., 2021). Regarding the inhibition of pathogenic microorganisms, TRQI components such as Huang-qin, Lian-qiao, Jin-yin-hua and bear bile powder show significant inhibitory effects against *S. aureus*, *Escherichia coli*, and *S. pneumoniae* (Cui et al., 2023; He et al., 2021; Hu et al., 2023) In addition, the baicalin present in Huang-qin has been shown to significantly inhibit the formation of bacterial biofilms in *Pseudomonas aeruginosa* (Liu et al., 2024) and *Klebsiella pneumoniae* (Han, 2022). During the progression of VAP, excessive release of inflammatory factors damages the alveolar-capillary barrier and impairs pulmonary surfactant production, a primary mechanism leading to acute respiratory distress syndrome (ARDS) (Zeng et al., 2021). The resulting inflammatory storm also affects other tissues and organs, resulting in a severe systemic inflammatory response and significantly increasing the incidence of other complications (Metersky and Kalil, 2018). Studies have shown that TRQI can modulate the immune response of pulmonary macrophages (Hu et al., 2022), enhance the body’s immune function, and attenuate the onset and progression of inflammatory responses. Specifically, metabolites such as kaempferol, luteolin, and quercetin in Lian-qiao can inhibit the release of inflammatory factors by regulating the NF-κB-mediated inflammatory pathway (Liu et al., 2016). Chlorogenic acid from Jin-yin-hua can suppress the development of acute lung injury by downregulating the STING signaling pathway (Xu et al., 2016). In addition, the tauroursodeoxycholic acid in bear bile powder can reduce vascular permeability, decrease inflammatory exudation, and attenuate hypoxic injury to alveolar-capillary endothelial cells (Liu et al., 2020).

#### 4.2.2 TCM theoretical mechanism of TRQI in treating VAP

The pathogenesis of VAP is complex due to the unique characteristics of the patient population. In TCM, the disease is generally considered to originate from a deficiency of zheng qi (vital energy), with phlegm, fire, heat, toxins, and blood stasis as manifestations. The disease presents with a combination of deficiency and excess. The pathological products, such as phlegm and stasis, generated during disease progression can further obstruct the body’s meridians, thereby exacerbating the condition (Huang, 2012). TRQI is composed primarily of, Lian-qiao, Jin-yin-hua, Huang-qin, Antelope horn, and bear bile powder. TRQI has the capacity to clear heat and detoxify the body while simultaneously resolving phlegm and removing stasis. In the treatment of VAP, TRQI functions by expelling pathogenic factors and regulating the generation and metabolism of pathological products within the body. In conjunction with contemporary medical interventions such as ventilators, which assist in restoring yang (vital energy), this approach replenishes zheng qi, eliminates heat toxins and phlegm-fire, and facilitates recovery.

### 4.3 Differences from previous studies

Previous meta-analyses have primarily focused on the duration of antibiotic use, length of ICU stay, and mortality rates, without analyzing inflammatory markers or the duration of ventilator use. Furthermore, a number of high-quality randomized controlled trials (RCTs) on this subject have been published in recent years. This study conducted a comprehensive search of the current published literature in both Chinese and English, including analyses of inflammatory markers in addition to the previously studied treatment durations. By integrating these aspects, this research offers a more complete evaluation of the efficacy and safety of the drug, providing a more detailed and enriched perspective than that offered by earlier studies.

### 4.4 Clinical and research implications

#### 4.4.1 Selection of clinical research indicators

Despite the existence of a multitude of clinical studies that have investigated the efficacy of TRQI in the treatment of VAP, the outcome indicators have predominantly concentrated on the analysis of inflammatory markers and the duration of treatment. However, the existing literature has not addressed all-cause mortality, which makes it challenging to assess the impact of TRQI on the prognosis of VAP patients. Moreover, only a limited number of studies have incorporated assessments of adverse reactions. It is recommended that future clinical research place greater emphasis on clinical prognosis indicators and safety evaluations, in order to provide a higher level of evidence for the clinical application of TRQI.

#### 4.4.2 Usage and course of TRQI in treating VAP

The pharmacological effects of TCM are contingent upon the dosage and manner of its administration. In previous studies, there has been a lack of consensus regarding the dosage and administration of TRQI. The findings of this study indicate that the majority of research studies utilized a dosage of 20–40 mL of TRQI diluted in an appropriate solution for intravenous infusion, resulting in favorable outcomes. The active ingredients of TCM are typically complex compounds that exert therapeutic effects after absorption, distribution, metabolism, and excretion. These processes often require time to manifest. The mean duration of TRQI treatment in this study was 10 days, which suggests that a treatment course exceeding 10 days may prove beneficial for the management of VAP.

### 4.5 Limitations of the study

1. Language Bias: The present study was limited to Chinese and English databases, which may have introduced a selection bias due to the exclusion of literature in other languages. 2. The heterogeneity of treatment plans is a further limitation of this study. The studies included in this review span the period from 2009 to 2023, during which time there have been developments in the treatment protocols and anti-infective medications used for VAP. 3. Publication bias: Studies with positive results are more likely to be published, which may overestimate the efficacy of TRQI. 4. The quality of the studies included in this review was assessed according to the criteria outlined in the methodology section. The quality assessment of some studies indicated moderate quality, with many studies failing to adequately address the importance of randomization and blinding methods. 5. The studies included in this analysis had relatively small sample sizes. A number of the included studies had small sample sizes, lacked sample size calculations, and were of moderate quality. As a result, the results should be interpreted with caution. 6. A further limitation of the studies was the lack of differentiation according to TCM syndrome. The lack of traditional Chinese medicine syndrome differentiation in some included studies limits the ability to provide targeted medication guidance for specific syndromes.

## 5 Conclusion

This study provides further evidence-based support for the clinical application of TRQI in the treatment of VAP. Additionally, it summarizes previous clinical research through a literature quality assessment, offering insights and recommendations for the design and implementation of future research protocols. The results of this study indicate that TRQI can improve inflammatory markers in VAP patients, reduce the duration of antibiotic and ventilator use, increase the weaning success rate, improve pulmonary infection scores, and shorten ICU stays. Nevertheless, further evidence is required to ascertain the safety profile with regard to adverse drug reactions. Moreover, some of the includeAccordinglyd clinical studies had relatively small sample sizes and lacked sample size calculations, the future study designs should be more rigorous to enhance the reliability of findings.

## Data Availability

The original contributions presented in the study are included in the article/Supplementary Material, further inquiries can be directed to the corresponding author.
