# Supplementary material for: Effects of Tanreqing injection against ventilator-associated pneumonia: a meta-analysis and systematic review of clinical studies
Source: Front Pharmacol. 2025 Mar 7;16:1545088. doi: 10.3389/fphar.2025.1545088 (PMC11925857; doi:10.3389/fphar.2025.1545088)
Supplement: Supplementary file 1 [file DataSheet2.docx]

The GRADE assessmen of outcome indicators

| Certainty assessment | | | | | | | | sample size | | Effect size | | Results | Importance |
| --- | --- | --- | --- | --- | --- | --- | --- | --- | --- | --- | --- | --- | --- |
| Outcome indicators | No. of studies | Study design | Risk of bias | Inconsistency | Indirectness | Imprecision | Other considerations | Test group | Control subjects | RR/MD | 95% CI |  |  |
| CRP | 12 | RCT | serious | not serious | not serious | not serious | 无 | 443 | 425 | MD=-19.04 | (-24.01，-14.07) | ⨁⨁⨁◯ Moderate | Critical |
| PCT | 9 | RCT | serious | not serious | not serious | not serious | 无 | 319 | 311 | MD=-0.54 | (-0.76，-0.32) | ⨁⨁⨁◯ Moderate | Critical |
| WBC | 6 | RCT | serious | not serious | not serious | serious | 无 | 188 | 186 | MD=-2.15 | (-3.01，-1.28) | ⨁⨁◯◯ Low | Critical |
| Duration of Antibiotic Use | 7 | RCT | serious | not serious | not serious | not serious | 无 | 253 | 250 | MD=-3.41 | (-4.04，-2.78) | ⨁⨁⨁◯ Moderate | Important |
| Duration of Ventilator Use | 8 | RCT | serious | not serious | not serious | not serious | 无 | 284 | 276 | MD=-2.90 | (-3.21，-2.59) | ⨁⨁⨁◯ Moderate | Important |
| Length of ICU Stay | 6 | RCT | serious | not serious | not serious | not serious | 无 | 213 | 211 | MD=-2.99 | (-3.80，-2.18) | ⨁⨁⨁◯ Moderate | Important |
| First Successful Weaning Rate | 4 | RCT | serious | not serious | not serious | serious | 无 | 188 | 175 | RR=1.19 | (1.07，1.32) | ⨁⨁◯◯ Low | Important |
| CPIS Score | 5 | RCT | serious | not serious | not serious | serious | 无 | 175 | 168 | MD=-1.76 | (-2.52，-1.00) | ⨁⨁◯◯ Low | Important |
