# Supplementary material for: Effects of Tanreqing injection against ventilator-associated pneumonia: a meta-analysis and systematic review of clinical studies
Source: Front Pharmacol. 2025 Mar 7;16:1545088. doi: 10.3389/fphar.2025.1545088 (PMC11925857; doi:10.3389/fphar.2025.1545088)
Supplement: Supplementary file 2 [file DataSheet1.docx]

The search strategies of Tanreqing injection for ventilator-associated pneumonia

| Databases | search strategies | Hit Counts |
| --- | --- | --- |
| Pubmed  (https://pubmed.ncbi.nlm.nih.gov/advanced/) | #1: All Fields= “Tanreqing Injection” OR “Tan re qing injection” OR “Tanreqing”  #2: All Fields= “ventilator-associated pneumonia” OR “pneumonia,ventilator-associated” OR “ventilator-associated pneumonitis”  #3: #1 and #2 | 0 |
| Web of science  (https://www.webofscience.com/) | #1: TS= “Tanreqing Injection” OR “Tan re qing injection” OR “Tanreqing”  #2: TS= “ventilator-associated pneumonia” OR “pneumonia,ventilator-associated” OR “ventilator-associated pneumonitis”  #3: #1 and #2 | 4 |
| Embase  (https://www.embase.com/#advancedSearch) | #1: Broad search= ‘Tanreqing Injection’ OR ‘Tan re qing injection’ OR ‘Tanreqing’  #2: Broad search= ‘ventilator-associated pneumonia’ or ‘pneumonia,ventilator-associated’ or ‘ventilator-associated pneumonitis’  #3: #1 and #2 | 2 |
| The Cochrane Library  (https://www.cochranelibrary.com/advanced-search) | #1: “Tanreqing Injection OR “Tan re qing injection” OR “Tanreqing”  #2: “ventilator-associated pneumonia” OR “pneumonia,ventilator-associated” OR “ventilator-associated pneumonitis”  #3: #1 and #2 | 0 |
| CNKI (https://www.cnki.net/) | SU%=（‘痰热清注射液’+‘痰热清’） AND SU%=（‘呼吸机’+‘呼吸机相关性肺炎’） | 46 |
| WanFang Data (https://w.wanfangdata.com.cn/) | 全部: ("痰热清注射液" or "痰热清") and 全部: ("呼吸机" or "呼吸机相关性肺炎") | 43 |
| VIP (http://www.cqvip.com/) | U=(痰热清注射液OR痰热清) and U=(呼吸机OR呼吸机相关性肺炎) | 42 |
| SinoMed (http://www.sinomed.ac.cn) | （"痰热清注射液"[全部字段:智能] OR "痰热清"[全部字段:智能])AND( "呼吸机"[全部字段:智能] OR "呼吸机相关性肺炎"[全部字段:智能]) | 31 |
